# Validation of onchocerciasis biomarker *N*-acetyltyramine-*O*-glucuronide (NATOG)

**DOI:** 10.1016/j.bmcl.2017.05.082

**Published:** 2017-08-01

**Authors:** Daniel Globisch, Lisa M. Eubanks, Ryan J. Shirey, Kenneth M. Pfarr, Samuel Wanji, Alexander Y. Debrah, Achim Hoerauf, Kim D. Janda

**Affiliations:** a Department of Chemistry, The Scripps Research Institute, 10550 North Torrey Pines Road, La Jolla, CA 92037, United States; b Department of Immunology, The Skaggs Institute for Chemical Biology, The Worm Institute of Research and Medicine (WIRM), The Scripps Research Institute, 10550 North Torrey, La Jolla, CA 92037, United States; c Institute of Medical Microbiology, Immunology, and Parasitology (IMMIP), University Hospital Bonn, Sigmund Freud Straße 25, 53105 Bonn, Germany; d Research Foundation in Tropical Diseases and Environment (REFOTDE), P.O Box 474, Buea, Cameroon; e Faculty of Allied Health Sciences, Kwame Nkrumah University of Science and Technology, Kumasi, Ghana; f Kumasi Centre for Collaborative Research in Tropical Medicine (KCCR), Kumasi, Ghana

**Keywords:** Onchocerciasis, Biomarker, Mass spectrometry, Metabolomics, Nematode

## Abstract

The Neglected Tropical Disease onchocerciasis is a parasitic disease. Despite many control programmes by the World Health Organization (WHO), large communities in West and Central Africa are still affected. Besides logistic challenges during biannual mass drug administration, the lack of a robust, point-of-care diagnostic is limiting successful eradication of onchocerciasis. Towards the implementation of a non-invasive and point-of-care diagnostic, we have recently reported the discovery of the biomarker *N*-acetyltyramine-*O*-glucuronide (NATOG) in human urine samples using a metabolomics-mining approach. NATOG’s biomarker value was enhanced during an investigation in a rodent model. Herein, we further detail the specificity of NATOG in active onchocerciasis infections as well as the co-infecting parasites *Loa loa* and *Mansonella perstans*. Our results measured by liquid chromatography coupled with mass spectrometry (LC-MS) reveal elevated NATOG values in mono- and co-infection samples only in the presence of the nematode *Onchocerca volvulus*. Metabolic pathway investigation of l-tyrosine/tyramine in all investigated nematodes uncovered an important link between the endosymbiotic bacterium *Wolbachia* and *O. volvulus* for the biosynthesis of NATOG. Based on these extended studies, we suggest NATOG as a biomarker for tracking active onchocerciasis infections and provide a threshold concentration value of NATOG for future diagnostic tool development.

## Graphical abstract

**Figure:**
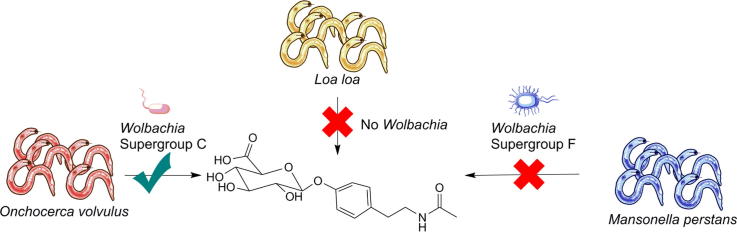

Onchocerciasis, also known as river blindness, is a parasitic disease, which imposes a massive burden on populations in areas with high poverty in developing countries. More than 95% of all infections are present in Central and Western Africa, where an estimated 37 million people in 31 Sub-Saharan countries are currently affected and an additional 100 million people continue to be at risk for infection by this Neglected Tropical Disease.^1^, ^2^, ^3^ Onchocerciasis is caused by the filarial nematode *Onchocerca volvulus* and is vector-transmitted by *Simulium* sp. black flies. *O. volvulus* infections can cause a myriad of severe effects on their human hosts including dermatitis, visual impairment and blindness. The WHO started the Onchocerciasis Control Programme in West Africa (OCP) in the 1970s to fight against this parasitic disease and to improve the quality of life of people living in these poverty-stricken areas.^1^, ^2^, ^3^ In 1995 a second phase was launched, the African Programme for Onchocerciasis Control (APOC), to increase the scope of this project and to further cover endemic African countries in Central Africa. In 2002, OCP ceased operations and many of the endemic regions were reorganized under APOC, which has managed and organized mass drug administrations (MDA) with the antiparasitic drug ivermectin (Mectizan®; donated from Merck and Co.) until the end of 2015^1^, ^2^, ^3^ and is now being continued by the Expanded Special Project for Elimination of Neglected Tropical Diseases (ESPEN) programme.^4^

Despite successes with the elimination of river blindness in parts of West Africa, two major goals still need to be achieved for controlling and ultimately eliminating onchocerciasis in other endemic regions through the APOC-Programme. First, a simple and robust point-of-care diagnostic test to replace the highly invasive and painful skin snip diagnostic test must be developed, which, if correctly designed, could also be used to monitor mass drug treatment progression.^5^, ^6^ Second, new drugs must be developed for macrofilarial elimination.^5^, ^6^ Currently, the skin snip remains the gold standard for diagnosing *O. volvulus* infections in the field. However, this procedure is largely rejected by communities due to its highly invasive nature. Furthermore, this method requires a long incubation period followed by counting of the microfilariae (MF) under a microscope. The final read out of this tedious and exasperating laboratory-based test suffers from low sensitivity and in some cases inaccurate analysis, in particular when people remain infected with fertile adult worms but have no skin MF due to frequent rounds of ivermectin MDA. Other diagnostics on the horizon are lacking selectivity or sensitivity as recently summarized in a review article by Vlaminck et al.^7^ Clearly, a more selective and point-of-care diagnostic would be beneficial for eradication programs focused on *O. volvulus* elimination.^7^, ^8^, ^9^

As a step towards the replacement of the skin snip diagnostic test for onchocerciasis, we previously reported the utility of our newly discovered biomarker *N*-acetyltyramine-*O*-glucuronide (NATOG), uncovered through a metabolomics-mining approach (Fig. 1).^10^ The importance of this finding is that non-protein based biomarkers, such as NATOG, can be readily integrated into a variety of diagnostics. The biosynthesis of this urine biomarker is proposed to arise from a combination of metabolic steps occurring in both the nematode and the human host. The nematode’s neurotransmitter tyramine derived from l-tyrosine is acetylated to form the inactivated metabolite *N*-acetyltyramine, which is secreted into the human host and subsequently glucuronidated. Glucuronidation is a metabolic clearance mechanism for exogenous metabolites in humans.

**Fig. 1:**
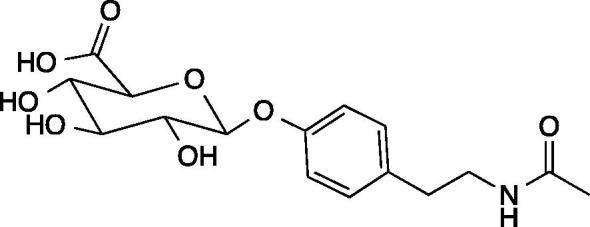
Chemical structure of biomarker *N*-acetyltyramine-*O*-glucuronide (NATOG).

We consider this metabolic link between the parasite and the human host as the basis for the selectivity of this metabolite for *O. volvulus* infections. Our previously reported results demonstrated that NATOG values are enhanced in samples from *O. volvulus* infected patients with respect to control samples. Additionally, a NATOG concentration dependence was observed, which allowed tracking of doxycycline and ivermectin treatments compared to placebo.^10^ The value of NATOG as a selective biomarker for onchocerciasis was further demonstrated using a model system where rodents were infected with the closely related nematode *Litomosoides sigmodontis*^11^ and increasing amounts of NATOG over the time course and progression of the infection were observed. Advantageously, this model system provided a direct correlation between NATOG concentrations and infection stages, which can be translated to the life cycle of *O. volvulus* in humans.

As an important step prior to implementation of a NATOG-based diagnostic for onchocerciasis, we sought to evaluate the disease/species-specificity of NATOG in human urine samples by analyzing a broader and more complex sample set. Quantitation of NATOG in urine samples collected from patients in areas where co-infections with other filarial parasites is common provided a challenging test for our onchocerciasis biomarker.

*Mansonella perstans* and *Loa loa* are two human filarial nematodes known to be co-endemic with *O. volvulus* in large parts of West and Central Africa.^12^, ^13^ *O. volvulus*-*M. perstans* (*Ov*/*Mp*) co-infections are common in areas such as the rain forest villages of Cameroon (up to 40% of participants studied) due to a similar epidemiology.^14^ *M. perstans* is a widespread human filarial parasite, transmitted through biting midges of the genus *Culicoides*.^15^ *M. perstans* infections cause in most cases only mild and not immediately detectable symptoms^14^; yet, an *M. perstans* infection can alter the host’s immune system thereby impacting the severity of other diseases including malaria, tuberculosis and HIV.^15^ From a therapeutic vantage, individuals co-infected with *M. perstans* can be safely treated with ivermectin.^16^

*O. volvulus*-*L. loa* (*Ov*/*Ll*) co-infections are less common than *Ov*/*Mp* co-infections, but are of high importance during MDA. Onchocerciasis-treatment with ivermectin in *Ov*/*Ll* co-infected patients can cause serious adverse events including neurological complications such as fatal encephalopathy.^6^, ^17^, ^18^ The reasons for these side effects are not fully clarified but have been linked to the density of *L. loa* MF.^18^ Therefore, it is imperative to be able to distinguish whether a patient is mono-infected with *O. volvulus* or whether a co-infection with *L. loa* is present.

To evaluate the significance of NATOG in samples from co-infected patients, we initiated our studies by establishing a NATOG concentration value indicative of an *O. volvulus* mono-infection. A large urine sample set consisting of both *O. volvulus* positive samples (N = 145) and *O. volvulus* negative control samples (N = 118) were analyzed using our established LC-MS method.^10^, ^11^ Precise quantification was achieved through the generation of a calibration curve using a deuterated NATOG analogue (D_3_-NATOG) as an internal standard (Supporting information/Fig. S1). The samples, collected in West African villages of Cameroon and Ghana, were initially diagnosed using standard procedures (skin snip and nodule palpitation) to identify them as either positive or negative for *O. volvulus* infections. Importantly, additional evaluations were conducted to exclude possible co-infections with the other filarial nematodes *M. perstans*, *L. loa* and *Wuchereria bancrofti*. The analysis of all *O. volvulus*-positive samples (N = 145) resulted in an average NATOG concentration of 42.8 ± 3.7 µM, slightly elevated from our previously reported *O. volvulus*-positive value of 36.9 ± 4.0 µM (Fig. 2/Fig. S2). Quantification of NATOG in all of the new *O. volvulus*-negative samples [c(NATOG) = 6.4 ± 0.7 µM] paralleled the former average concentration of 7.0 ± 2.7 µM. Overall, the results obtained from these latest data sets, further strengthen our finding that NATOG concentrations are significantly increased in *O. volvulus* infected individuals compared to non-infected individuals (*P* < 0.0001).

**Fig. 2:**
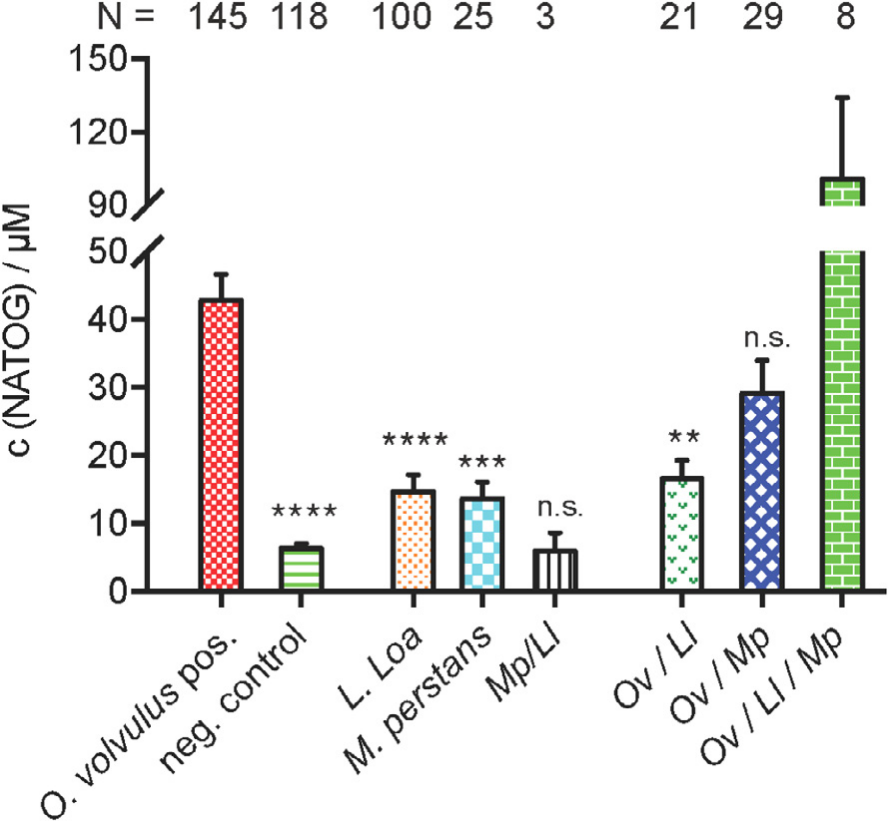
Quantification of NATOG in human samples. Average values are presented with SEM. Data were analyzed by ANOVA, followed by Tukey’s multiple comparison method to compare onchocerciasis with each sample. See Fig. S2 for Tukey’s box-and-whisker plot for this data set.

Now that a NATOG value signifying an *O. volvulus* mono-infection has been established, we next analyzed samples collected in Cameroonian villages from patients that were mono-infected with either *L. loa* (N = 100) or *M. perstans* (N = 25). These samples were diagnosed by microscopy, i.e. *L. loa* and *M. perstans* MF were directly analyzed in blood smears and on filters.^19^ Samples were also examined to confirm the absence of a third potential co-infecting filarial parasite common in these areas, *W. bancrofti*, and in all cases the patient was determined to be negative for this particular nematode. NATOG concentrations in both sample sets were determined by LC-MS to be significantly reduced compared to *O. volvulus* mono-infected samples with average NATOG values of 14.7 ± 2.5 µM (±SEM; P < 0.0001) and 13.6 ± 2.5 µM (±SEM; P < 0.0002) in patients with *L. loa* and *M. perstans* mono-infections, respectively (Fig. 2). These new findings further validate the statistical significance and specificity of NATOG as a measure for positive *O. volvulus* mono-infections over other parasitic diseases.

As described, *vide supra*, *M. perstans* and *L. loa* are co-endemic with *O. volvulus*, which represents a key test for NATOG’s specificity. A collection of 61 samples from patients diagnosed to be co-infected with at least two parasites were analyzed and the concentration of NATOG present in each sample was quantified. The samples with a dual co-infection consisting of *M. perstans* and *L. loa*, *Mp/Ll*, (N = 3) yielded the lowest average NATOG value of 6.0 ± 2.7 µM (± SEM), as expected in the absence of *O. volvulus*. Due to the limited sample number, no statistical significance analysis was feasible. *O. volvulus* dual co-infection samples *Ov*/*Ll* (N = 21) and *Ov*/*Mp* (N = 29) were quantified as c(NATOG) = 16.6 ± 2.8 µM (±SEM) and c(NATOG) = 29.2 ± 4.8 µM (±SEM), respectively. Importantly, statistical significance analysis yielded a clear distinction between the *O. volvulus*-positive and the *Ov*/*Ll*-sample values (p < 0.002). This is a key finding because severe side effects can be caused by the ivermectin treatment of *O. volvulus*-positive patients that are co-infected with *L. loa*; similar adverse reactions have not been reported for the case of *O. volvulus*-positive patients co-infected with *M. perstans*. Our final sample set came from individuals (N = 8) infected with all three nematodes (*O. volvulus*, *L. loa* and *M. perstans*). Surprisingly, a very high NATOG average concentration of c(NATOG) = 100.5 ± 33.5 µM (±SEM) was observed. However, this result needs to be viewed with caution as the sample set was small and thus further investigations will be necessary to confirm or refute this effect.

The data accumulated from these sample sets was then examined collectively to probe how NATOG quantification could be used to implement a field assay for onchocerciasis diagnosis. To start, we pooled NATOG concentration values from all *O. volvulus-*positive samples and grouped NATOG values from this study and our previous study accordingly (details are summarized in the Supporting information).^10^ All NATOG values from mono-infections with *O. volvulus* were combined in the first column (Fig.3*a*) and displayed as an average concentration of c(NATOG) = 40.3 ± 2.7 µM (±SEM; N = 240). In the second column, values from all three co-infections with *O. volvulus* (*Ov*/*Ll*, *Ov*/*Mp*, and *Ov*/*Ll/Mp*) were combined with *O. volvulus* mono-infection values resulting in a similar average NATOG concentration value of 39.2 ± 2.5 µM (±SEM; N = 298). Lastly, all samples without the detectable presence of *O. volvulus* (*O. volvulus*-negative control, *L. loa* mono-infection, *M. perstans* mono-infection, *L. loa*/*M. perstans* co-infection, and *Lymphatic filariasis*) were combined and resulted in an average concentration of c(NATOG) = 9.29 ± 0.95 µM (±SEM; N = 302). This overall sample analysis illustrates the selectivity of NATOG for active onchocerciasis-infections and demonstrates that the presence of *O. volvulus* is required for elevated NATOG levels.

**Fig. 3:**
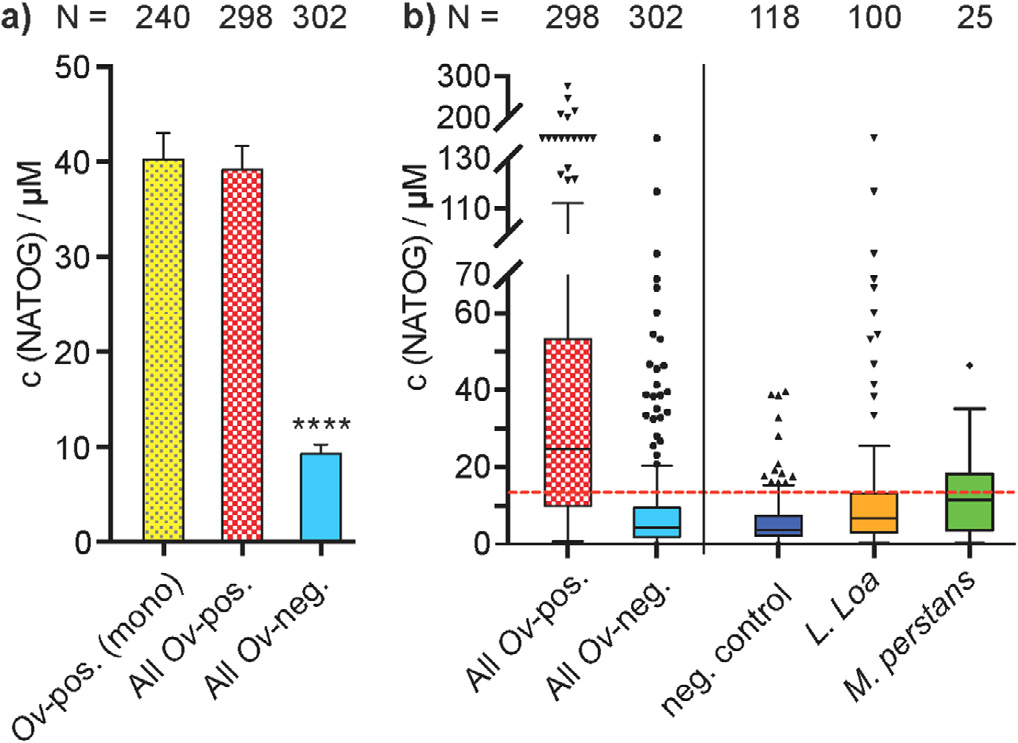
a) Comparison of average NATOG concentration in sample with and without the presence of *O. volvulus*. Error bars represent SEM values. Statistical significance was calculated using an unpaired *t*-test with Welch’s correction. b) Tukey’s box-and-whisker plot with median values for NATOG. The suggested threshold for a diagnostic test of 13 µM is labeled in red ().

Further clarification on the NATOG concentration distribution is depicted in Fig.3b. This box plot illustrates the median values of our quantified data for selected sample sets. The median concentration of all *O. volvulus* positive samples was found to be 24.8 µM. This NATOG value is highly elevated over all other sample sets with a value of 4.4 µM for all combined *O. volvulus* negative samples, 3.6 µM for uninfected negative control samples, 6.8 µM for *L. loa* mono-infections and 11.4 µM for *M. perstans* mono-infections. The measured high value in the “all *O. volvulus* negative control samples (All Ov-neg)” stems from the inclusion of the *L. loa* mono-infection positive samples. Taking into account the statistical analysis of all our quantified data, we suggest a NATOG threshold value of 13 µM for diagnosis of an active *O. volvulus* infection. All geometric mean and median NATOG values for each sample group except “All Ov-pos.” are below this threshold. In all cases the 95% confidence intervals of the geometric mean analysis are below this value including the crucial *L. loa*-positive sample group (Fig. S3 and Table S1). Moreover, devices grounded upon this NATOG level would present a mere 13.6% of the neg. control sample group as false-positive.

There is a clear distinction observed between the levels of NATOG present in samples collected from patients with an *O. volvulus* infection versus a *L. loa* or *M. perstans* infection. The reason behind these variations remains an intriguing question. One possible explanation may lie in differences in the nematode’s metabolic pathways and/or regulation of these pathways, especially with respect to l-tyrosine and tyramine. Biosynthesis of *N*-acetyltyramine, the deactivated metabolite that gets excreted into the human host for glucuronidation, is thought to proceed through a two-step enzymatic process in *O. volvulus*.^10^ First, l-tyrosine undergoes decarboxylation by a tyrosine decarboxylase (TDC) enzyme to yield tyramine followed by *N*-acetylation by an *N*-acetyltransferase (NAT) to form *N*-acetyltyramine (Fig. 4a).^10^, ^11^ Completion of the genome sequence of *O. volvulus* from a Cameroon isolate (BioProject PRJEB513) allows for the analysis of the genetic information from *O. volvulus*.^20^ Annotation of the genome sequencing data resulted in the identification of a general amino acid decarboxylase (Ovo-tdc-1, OVOC10783), an ortholog of the *C. elegans* tyrosine decarboxylase gene (*tdc–1*), and recent transcriptome and proteome analyses have indicated the presence of this protein in all developmental stages of *O. volvulus* (microfilariae, L2, L3 larvae, and adult worms).^21^ Not surprisingly, a highly homologous protein can be found in a Cameroon isolate of *L. loa* (LOAG_07708; EFO20782.2; BioProject PRJNA60051) as well as several other filarial nematodes known to infect humans including *W. bancrofti*, *B. malayi*, and *B. timori* (Fig. S4).^22^ Unfortunately, analysis of the parasitic nematodes from the genus *Mansonella* (*M. perstans*, *M. streptocerca*, and *M. ozzardi*) is not possible due to the lack of genome sequencing information at this juncture. However, as previously reported, the rodent parasite *L. sigmodontis* used in *O. volvulus* infection model systems contains a decarboxylase enzyme similar to the human parasite’s protein based on amino acid sequence alignments.^11^

**Fig. 4:**
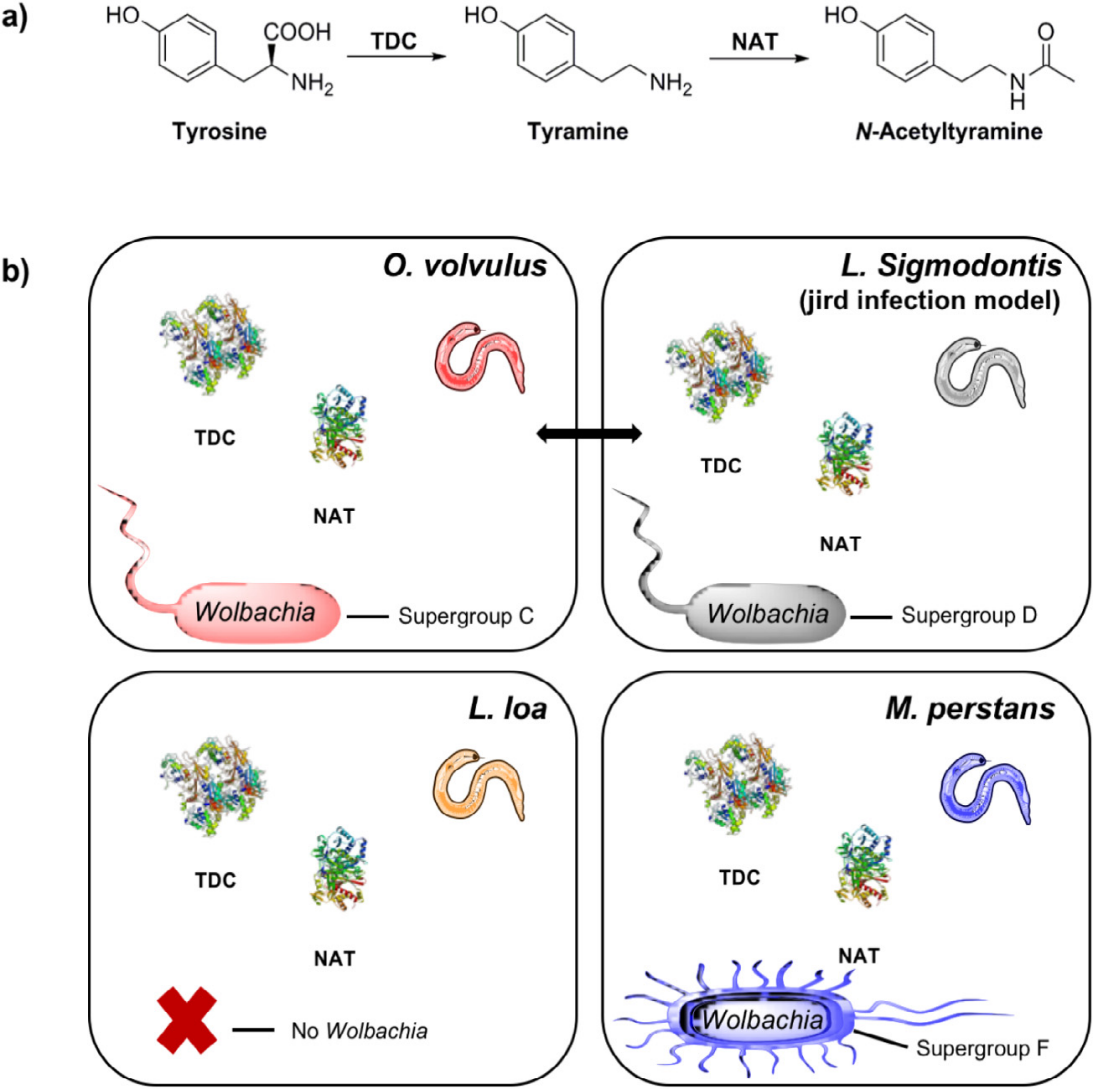
Biosynthetic analysis. a) Nematode two-step enzymatic conversion of tyrosine to *N*-acetyltyramine, the precursor for NATOG production in *O. volvulus* infected individuals. b) Schematic representation illustrating the similarities and differences found among the filarial nematodes *O. volvulus*, *L. loa*, and *M. perstans* influencing NATOG concentration levels observed in their human hosts; an analogous depiction for the rodent filarial nematode, *L. sigmodontis*, used in *O. volvulus* infection models. TDC – tyrosine decarboxylase; NAT – *N*-acetyltransferase.

The widespread presence of decarboxylase enzymes among nematodes, suggests that the initial step in the formation of NATOG likely proceeds through a common pathway in these organisms (Fig.4). The second metabolic reaction, *N*-acetylation of tyramine, is thought to be catalyzed by an *N*-acetyltransferase type enzyme and we have previously suggested two hypothetical proteins, OVOC6155 and OVOC3302, found in *O. volvulus*^11^; a third additional uncharacterized protein OVOC9342 may also be a candidate based on the most recently published genomic data and analysis.^21^ While the role of these proteins in the transformation of tyramine to *N*-acetyltyramine for NATOG biomarker production is still speculative, database searches have again revealed highly homologous counterparts in other related filarial nematodes, including *W. bancrofti, B. malayi*, *B. timori*, and more importantly *L. loa* and *L. sigmodontis* (Figs. S5–S7). The compilation of these findings does not necessarily preclude the notion that variations in the production of NATOG may occur at a biosynthetic level, however, an alternative explanation may be warranted.

Therefore, we shifted our focus to the obligate endosymbiotic bacterium *Wolbachia*, which infects the majority of filarial nematodes that in turn infect humans. Studies have revealed that *Wolbachia* is necessary for nematode reproduction and survival, plays a central role in nematode pathogenicity, and provides essential metabolic supplementation to their filarial host.^23^, ^24^, ^25^ One major dissimilarity between *O. volvulus* and *L. loa* is that *O. volvulus* is *Wolbachia*-dependent, whereas *L. loa* is naturally *Wolbachia*-free (Fig.4b).^26^, ^27^, ^28^, ^29^, ^30^ In the case of *M. perstans*, this nematode is host to the supergroup F *Wolbachia* strain, which is phylogenetically distinct from the supergroup C/D endosymbionts normally present in filarial nematodes including *O. volvulus* (supergroup C) and *L. sigmodontis* (supergroup D).^31^, ^32^, ^33^ One additional *Wolbachia*-related aspect having the potential to separate *O. volvulus* infections from other infections is that a predicted *N*-acetyltransferase protein has been identified in the *Wolbachia* endosymbiont of *O. volvulus* str. Cameroon (BioProject PRJEB4840, WP_025263975.1), which may contribute to NATOG production.^20^ In total, the presence or absence of *Wolbachia*, the distinction of *Wolbachia* supergroups (C/D versus F), and the identity of a putative *N*-acetyltransferase in *Wolbachia* from *O. volvulus* could all influence the differences observed in the levels of NATOG among nematode infected patients. Additionally, these new insights in the biosynthesis of NATOG highlight the similarity between *O. volvulus* and *L. sigmodontis* with respect to *Wolbachia*. In combination with our previous study these similarities support the notion that the *L. sigmodontis*-jirds infection model is a promising test system for *O. volvulus*.^11^

We submit our new data analysis further strengthens NATOG as a biomarker for onchocerciasis monitoring and demonstrates the importance of both sample selection and analysis. We make this latter statement in reference to a recently published study by Lagatie et al. contesting the potential of NATOG to successfully monitor human field treatments with ivermectin in Ghana.^34^ From our presented data, *vide supra*, we would expect only minimal amounts of NATOG to be present in these post-treated patients, which were subject to MDA programs (0–10 times). Thus, from the Lagatie study samples were determined to be *O. volvulus*-positive based upon nodule palpitation. Using this as their initial metric MF-counting of these nodule-positive samples presented low MF-counts (99% below 5 MF/mg).^34^ While 89% of these samples were MF-negative, which supports successful ivermectin treatment. In support of this, the reported NATOG values in the study from Lagatie et al. were in the range of our *O. volvulus*-negative control samples (including 51 samples collected from patients infected with *Lymphatic filariasis*).^34^ Moreover, these results parallel our published NATOG values stemming from samples containing combined ivermectin and doxycycline-treatment.^10^ While NATOG as a biomarker could not be fully exhibited in the Lagatie study, we submit that their findings demonstrate that quantification of NATOG can be used for monitoring patient treatment progression, which is a clear advantage over the analysis of MF in skin snips.

In conclusion, our data validate both the specificity and concentration dependence of NATOG in an active onchocerciasis infection. Genes encoding both classes of relevant nematode enzymes for NATOG biosynthesis, decarboxylases and *N*-acetyltransferases, have been identified in *O. volvulus* and presence of the corresponding proteins have been noted throughout its life cycle.^20^, ^21^ We propose a concentration of 13 µM NATOG as a cut-off for the presence of viable *O. volvulus* parasites. NATOG, as a biomarker for *O. volvulus* infection monitoring is not “perfect” as there appears to be some cross-over interference from co-infected samples involving *L. loa*. However, we would counter that parallel tests are still required for all currently developed and used diagnostics for *O. volvulus* and that no singular diagnostic exists that can selectively analyze all parasites in a simultaneous fashion.^35^ Thus, addition of a second biomarker for *L. loa* is already required to map this infection so that ivermectin MDA is not introduced into areas co-endemic for both infections. Based on our success with *O. volvulus*, a metabolomics analysis approach of other nematodes provides a powerful strategy for the discovery of small molecule biomarkers that may be added as an additional test with NATOG. Alternatively, a NATOG test can be combined with tests based on other analysis strategies such as reported transcriptome and proteome analysis.^10^, ^36^, ^37^ Still even with this caveat, NATOG should be vigorously considered as another metric to be added to the arsenal for the field monitoring of active *O. volvulus* infections.
