# Supplementary material for: Validation of onchocerciasis biomarker N-acetyltyramine-O-glucuronide (NATOG)
Source: Bioorg Med Chem Lett. 2017 Aug 1;27(15):3436–40. doi: 10.1016/j.bmcl.2017.05.082 (PMC5510726; doi:10.1016/j.bmcl.2017.05.082)
Supplement: Supplementary data [file mmc1.pdf]

SUPPLEMENTARY MATERIAL

Validation of onchocerciasis biomarker *N*-acetyltyramine-*O*-glucuronide (NATOG)

Daniel Globisch[a,b,*], Lisa M. Eubanks[a,b], Ryan J. Shirey[a,b], Kenneth M. Pfarr[c], Samuel Wanji[d], Alexander Y. Debrah[e,f], Achim Hoerauf[c], and Kim D. Janda[a,b]

[a] *Department of Chemistry, The Scripps Research Institute, 10550 North Torrey Pines Road, La Jolla, CA 92037, United States*
[b] *Department of Immunology, The Skaggs Institute for Chemical Biology, The Worm Institute of Research and Medicine (WIRM), The Scripps Research Institute, 10550 North Torrey, La Jolla, CA 92037, United States*
[c] *Institute of Medical Microbiology, Immunology, and Parasitology (IMMIP), University Hospital Bonn, Sigmund Freud Straße 25, 53105 Bonn, Germany*
[d] *Research Foundation in Tropical Diseases and Environment (REFOTDE), P.O Box 474, Buea, Cameroon*
[e] *Faculty of Allied Health Sciences, Kwame Nkrumah University of Science and Technology, Kumasi, Ghana*
[f] *Kumasi Centre for Collaborative Research in Tropical Medicine (KCCR), Kumasi, Ghana*
[*] *Corresponding author at: Science For Life Laboratories, Department of Medicinal Chemistry, Division of Analytical Pharmaceutical Chemistry, Uppsala University, Husargatan 3, 751 23 Uppsala, Sweden*

Overview

Human urine samples 2
Ethics statement 2
Sample preparation and metabolite extraction 3
LC-ESI-TOF-MS analysis 3
Calibration curve and statistical analysis 4
Figures S1-S7 5
Table S1 15
References 16

Human urine samples

Archived human urine from *O. volvulus* infected and uninfected volunteers was collected as part of a European Union Seventh Framework Programme Research grant, contract 131242 “Enhanced Protective Immunity Against Filariasis” (EPIAF). Infected individuals were those with palpable nodules and microfilaridermia by skin snip.[1, 2] Uninfected individuals were defined as persons with no palpable nodules and microfilariae negative skin snips.

Archived human urine from volunteers infected with *Loa loa*, *Masonella perstans* and dual or triple infections with *O. volvulus* were collected as part of the RADIO Consortium supported by a grant to the Institute of Microbiology, Immunology and Parasitology, University Hospital of Bonn from the Bill & Melinda Gates Foundation (OPP1083888). Infections were confirmed by microscopic examination of blood-borne microfilariae as previously described.[3-5]

Ethics statement

Ghana: Ethical clearance for the use of archived samples for biomarker research was obtained from the Committee on Human Research Publication and Ethics at the University of Science and Technology in Kumasi, Ghana.

Cameroon: Ethical clearance for the use archived samples for biomarker research was obtained from the National Institutional Review board, Yaoundé and Administrative clearance from the Delegation of Public Health, South West Region. Approval for the study was granted by the National Ethics Committee of Research for Human Health in Cameroon.

Germany: Approval of the use of archived samples was obtained from the Ethics Committee at the University Hospital of Bonn, Germany.[3]

All studies and sample collections were conducted in accordance with the Helsinki Declaration of 1975 as revised in 1983, 2000 and 2002.

Sample preparation and metabolite extraction

Solvents used were of HPLC grade. A methanol precipitation of proteins was conducted by adding 400 µl of ice-cold methanol to 100 µl aliquots of urine samples spiked with a known concentration of isotopic labeled internal standard **D_3-NATOG**. The samples were vigorously shaken for 30 s and allowed to rest on ice for 30 min. After centrifugation at 13,780 g for 5 min, the metabolite containing supernatant was removed from the precipitated protein pellet and transferred to fresh tubes. The supernatant samples were dried in a GeneVac EX-2 Evaporation System (GeneVac Inc., Valley Center, New York, USA) at ambient temperature and then resuspended to a volume of 50 µl in water/acetonitrile (95/5), vigorously shaken for 30 s and then centrifuged again at 13,780 g for 5 min. After being transferred to pre-labeled LC vials, samples were stored at 4 °C and transferred to the LC-MS thermostated autosampler cooled to 8 °C.

LC-ESI-TOF-MS analysis

The samples (8 µl injection volume) were analyzed with an electrospray-ionization time-of-flight mass spectrometry (ESI-TOF-MS) (Agilent TOF 6210, Agilent Technologies, Santa Clara, CA, USA) and chromatographic separation by HPLC (Agilent 1200 LC, Agilent Technologies, Santa Clara, CA, USA) with a flow rate of 70 µl/min using a T3 Atlantis column (3 mm, 1.0 mm × 150 mm; Waters Corporation, Milford, MA, USA). The column temperature was maintained at 30 ºC. Eluting buffers were buffer A (0.1% HCOOH in H_2O) and buffer B (0.1% HCOOH in MeOH). Gradient: 0 → 4 min; 2% → 2% buffer B; 4 → 25 min; 2% → 95% buffer B; 25 → 30 min; 95% → 2% buffer B; 30 → 33 min; 2% buffer B; 33 → 58 min; 2% → 2% buffer B. The chromatographic eluent was directly injected into the ion source without prior splitting. Data were collected in positive electrospray ionization (ESI) mode scanning in centroid mode from 200-1200 m/z with a scan rate of 1.0 spectrum per second in 2 GHz extended dynamic range. The capillary voltage was 3,500 V; the nebulizer pressure, drying gas flow and gas temperature were set to 20 psig, 7 l/min and 350 °C, respectively. Parameters of the mass spectrometer were tuned prior each sample set with the Agilent ESI tune mix for TOF systems (Agilent Technologies, Santa Clara, CA, USA). Details on mass spectrometric quality control have previously been reported.[6]

Calibration curve and statistical analysis

Mass calibration curves for the labeled (**D_3-NATOG**) and the natural synthesized compound (**NATOG**) were obtained at five different concentration ratios. For each concentration ratio, an average value of three independent measurements was determined (*Fig. S1*). The labeled compound was mixed with three independent prepared solutions of the unlabeled molecule **NATOG**. The areas of labeled and unlabeled nucleosides of the LC-MS measurements were determined using Mass Hunter Qualitative Analysis software version B.03.01 (Agilent Technologies, Santa Clara, CA, USA) by extraction of the specific ion current with a mass filter of m/z = 356.1200-356.1600 for **NATOG** and m/z = 359.1400-359.1800 for **D_3-NATOG**. The linear fit of the determined area ratios over the concentration ratios resulted in a perfect R^2-value of 1.0000. Quantification was performed with addition of the isotopic labeled compound **D_3-NATOG** at a known concentration to each urine sample (50-100 µl) and the area of the isotopic labeled over natural compound was determined by integration. The exact concentration of **NATOG** was calculated using the obtained area ratio and the linear equation of the calibration curve. Statistical analysis of the quantified data in *Figs. 2, 3, S2, S3* and *Table S1* was performed using GraphPad Prism, version 7 for Mac OS X (GraphPad Software).

Figures S1-S7

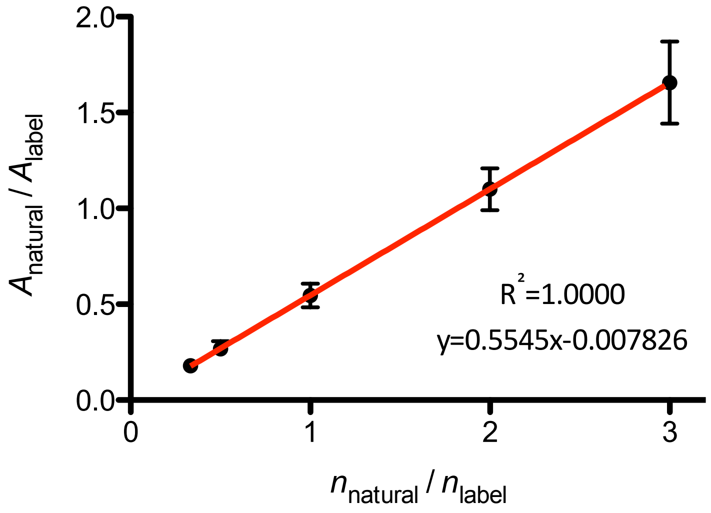

Figure S1. Calibration curve utilized for quantification of NATOG in urine samples.

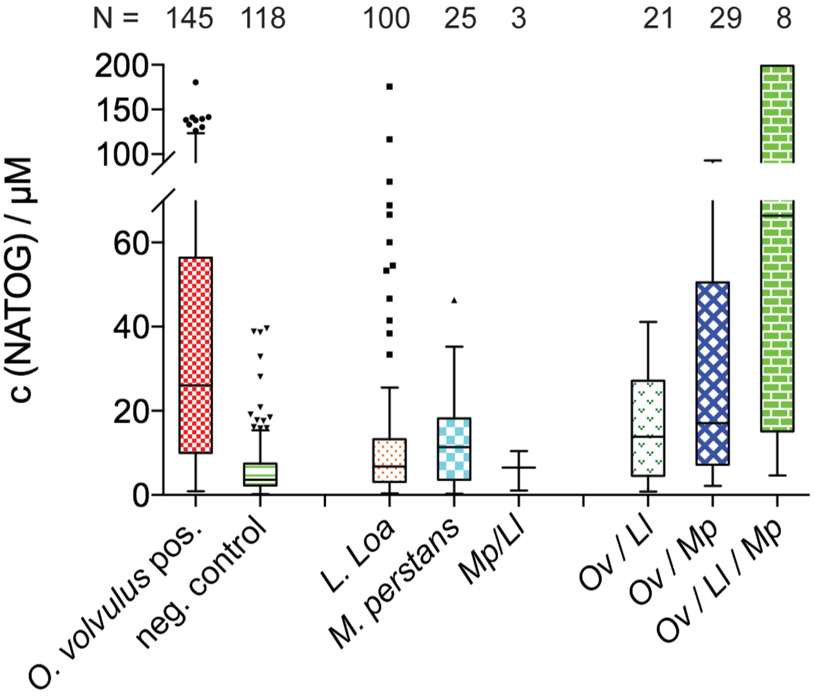

Figure S2. Tukey's box-and-whisker plot with median values for the same data set as in *Figure 2*.

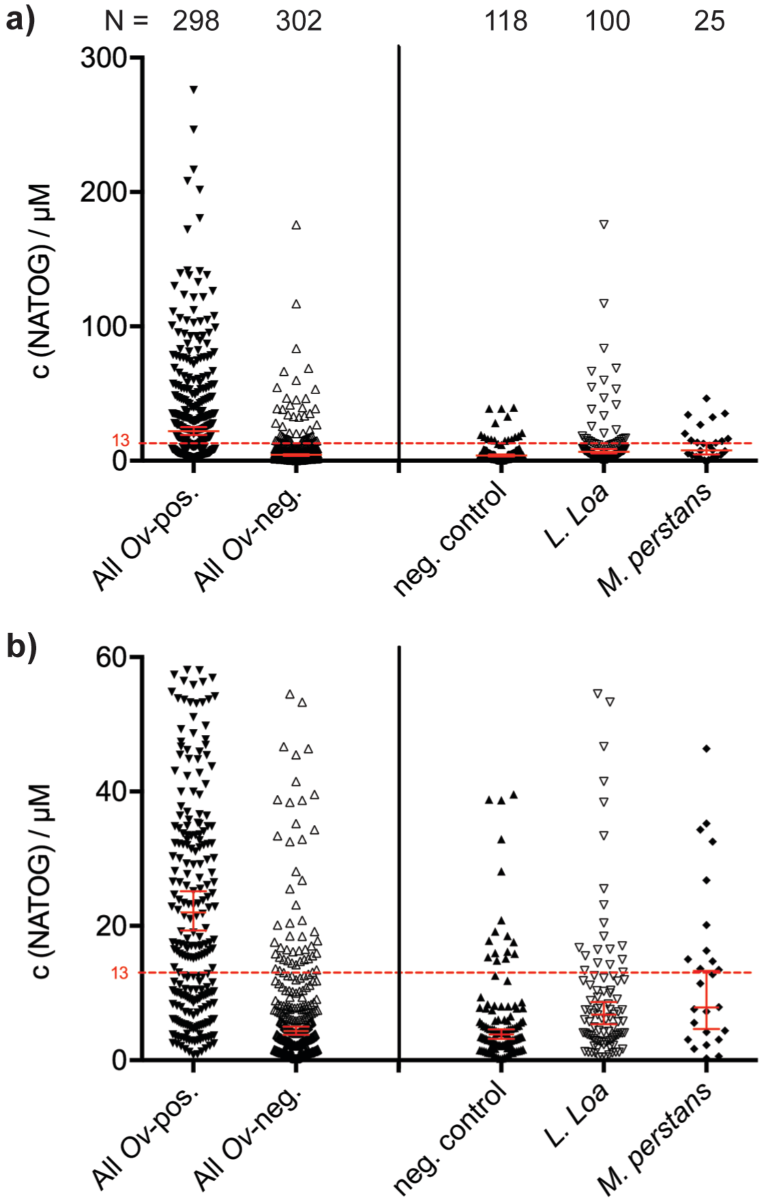

Figure S3. Scatter dot plot presentation of NATOG values with geometric mean and a 95% confidence interval. a) All measured data points; b) Zoom into relevant data points between 0 and 60 µM close to the suggested threshold for a diagnostic test of 13 µM (---).

```
1                                              50
      B. malayi    (1) MESKKWQPKKTGGMSSTEFRKYGKEVVDYIADYIENIQKRRVVPAIEPGY
    W. bancrofti   (1) -------------MSSTEFRKYGKEVVDYIADYIENIQKRRVVPAIEPGY
      B. timori    (1) ----------------TEFRKYGKEVVDYIADYIENIQKRRVVPTIEPGY
         L. loa    (1) MENKTWSPKKTAGMSSTEFRKYGKEVVDYIADYIENIQKRRVVPAIEPGY
 L. sigmodontis    (1) METKISPPKKTSGMSSTEFRKYGKEVVDYIADYIENIQKRRVVPAIEPGY
     O. volvulus   (1) -------------MDISEFRKFGKELVDYIADYIENIQKRRVVPSIEPGY
      Consensus    (1) ME K    PKKT GMSSTEFRKYGKEVVDYIADYIENIQKRRVVPAIEPGY
                       51                                              100
      B. malayi   (51) LRNLLPNMAPQHAEAFEDVISDFDRYIMPGITHWQHPRFHAYFPAGNAFP
    W. bancrofti  (38) LRNLLPDMAPQHAEAFEDVISDFDRYIMPGVTHWQHPRFHAYFPAGNAFP
      B. timori   (35) LRNLLPNMAPQHAEAFEDVISDFDRYIMPGITHWQHPRFHAYFPAGNAFP
         L. loa   (51) LRNLLPDMAPQNAEAFEDVINDFDKYIMPGITHWQHPRFHAYFPAGNAFP
 L. sigmodontis   (51) LRDLLPDVAPQHAEAFEDVINDFDKFIMPGITHWQHPRFHAYFPAGNAFP
     O. volvulus  (38) LRDLLPGVAPQHAESFKDVINDFDKYIMPGVTHWQHPRFHAYFPAGNAFP
      Consensus   (51) LRNLLPDMAPQHAEAFEDVISDFDKYIMPGITHWQHPRFHAYFPAGNAFP
                       101                                             150
      B. malayi  (101) NLLADMISDAIGAVGFSWAACPAMTELEIIMLDWFGRMIGLPDAFLPFTE
    W. bancrofti  (88) NLLADMISDAIGAVGFSWAACPAMTELEIIMLDWFGRMIGLPDAFLPFTE
      B. timori   (85) NLLADMISDAIGAVGFSWAACPAMTELEIIMLDWFGRMIGLPDAFLPFTE
         L. loa  (101) NLLADMISDAIGAVGFSWAACPAMTELEIIMLEWLGRMIGLPDAFLPFTE
 L. sigmodontis  (101) NLLADMISDAIGAVGFSWAACPAMTELEIIMLDWLGRMVGLPDAFLPFTE
     O. volvulus  (88) NIFADMLSDAIGAIGFSWAACPAMTELEIIMLDWLGRMLGLPEAFLPFTE
      Consensus  (101) NLLADMISDAIGAVGFSWAACPAMTELEIIMLDWLGRMIGLPDAFLPFTE
                       151                                             200
      B. malayi  (151) NGKGGGVIQGSASECNFVSLLAARFEVLKELRQRFPFVEEGLLLSKLVAY
    W. bancrofti (138) NGKGGGVIQGSASECNFVSLLAARFEVLKELRQRFPFVEEGLLLSKLVAY
      B. timori  (135) NGKGGGVIQGSASECNFVSLLAARFEVLKELRQRFPFVEEGLLLSKLVAY
         L. loa  (151) NGKGGGVIQGSASECNFVSLLAARFEVLKELRQRFPFVEEGLLLSRLVAY
 L. sigmodontis  (151) NGKGGGVIQGSASECNFVSLLVARFEMLKELRQRFPFVEEGLLLSKLVAY
     O. volvulus (138) NGKGGGVIQGSASECNFVSLLAARFEVLKELRHRFPFVDEGLLLSKLVAY
      Consensus  (151) NGKGGGVIQGSASECNFVSLLAARFEVLKELRQRFPFVEEGLLLSKLVAY
                       201                                             250
      B. malayi  (201) CSKEAHSSVEKACMIGMVKLKILDTDTKFRLRGKTLRLAIEEDRNLGLIP
    W. bancrofti (188) CSKEAHSSVEKACMIGMVKLKILDTDAKFRLRGRTLRLAIEEDRNLGLIP
      B. timori  (185) CSKEAHSSVEKACMIGMVKLKILDTDAKFRLRGKTLRLAIEEDRNLGLIP
         L. loa  (201) CSKEAHSSVEKACMIGMVKLKILETDTKFRLRGQTLRLAIEEDRNLGLIP
 L. sigmodontis  (201) CSKEAHSSVEKACMIGMVKLKILDTDAKFRLRGKTLSSAIEEDRNLGLIP
     O. volvulus (188) CSKESHSSVEKACMIAMVKLKILDTDSKFRLRGETLRLAIEEDRDLGLIP
      Consensus  (201) CSKEAHSSVEKACMIGMVKLKILDTDAKFRLRGKTLRLAIEEDRNLGLIP
```

```
                           251                                              300
        B. malayi   (251)  FFVSTTLGTTSCCSFDVLSEIGPVCHENDLWLHVDGAYGGSAMICPEFRP
      W. bancrofti  (238)  FFVSTTLGTTSCCSFDVLSEIGPVCHENDLWLHVDGAYGGSAMICPEFRP
        B. timori   (235)  FFVSTTLGTTSCCSFDVLSEIGPVCHENDLWLHVDGAYGGSAMICPEFRP
           L. loa   (251)  FFVSTTLGTTSCCSFDVLSEIGPVCQENDLWLHVDGAYGGSAMICPEFRP
   L. sigmodontis   (251)  FFVSTTLGTTSCCSFDVLSEIGPVCKENDLWLHVDGAYGGSAMICPEFRP
       O. volvulus  (238)  FFVSTTLGTTSCCSFDVLSEIGPVCQKNDLWLHVDGAYGGSAMICPEFRP
        Consensus   (251)  FFVSTTLGTTSCCSFDVLSEIGPVCHENDLWLHVDGAYGGSAMICPEFRP
                           301                                              350
        B. malayi   (301)  LMEGIEYAMSFNTNPNKFMLINFDCSTMWVKDRYKLTQALVVDPLYLQHS
      W. bancrofti  (288)  LMEGIEYAMSFNTNPNKFMLINFDCSTMWVKDRYKLTQALVVDPLYLQHS
        B. timori   (285)  LMEGIEYAMSFNTNPNKFMLINFDCSTMWVKDRYKLTQALVVDPLYLQHS
           L. loa   (301)  LMEGIEYAMSFNTNPNKFMLINFDCSTMWVKDRYKLTQALVVDPLYLQHS
   L. sigmodontis   (301)  LMEGIEYAMSFNINPNKFMLINFDCSTMWVKDRYKLTQALVVDPLYLQHS
       O. volvulus  (288)  LMDGIEYAMSFNTNPNKFMLINFDCSTMWVKDRYKLTQALVVDPLYLQHS
        Consensus   (301)  LMEGIEYAMSFNTNPNKFMLINFDCSTMWVKDRYKLTQALVVDPLYLQHS

                           351                                              400
        B. malayi   (351)  WTDKAIDYRHWSIPLSRRFRSLKLWFVIRVYGVEGLQSYIRRHCRLAKLF
      W. bancrofti  (338)  WTDKAIDYRHWSIPLSRRFRSLKLWFVIRVYGVEGLQSYIRRHCRLAKLF
        B. timori   (335)  WTDKAIDYRHWSIPLSRRFRSLKLWFVIRVYGVEGLQSYIRRHCRLAKLF
           L. loa   (351)  WTDKAIDYRHWSIPLSRRFRSLKLWFVIRVYGVNGLQNYIRRHCRLAKLF
   L. sigmodontis   (351)  WTDKAIDYRHWGIPLSRRFRSLKLWFVIRVYGVEGLQNYIRKHCRLAKLF
       O. volvulus  (338)  WTDKAIDYRHWGIPLSRRFRSLKLWFVIRVYGVEGLQNYIRKHCYLAKLF
        Consensus   (351)  WTDKAIDYRHWSIPLSRRFRSLKLWFVIRVYGVEGLQSYIRRHCRLAKLF
                           401                                              450
        B. malayi   (401)  EQLIRADNIFEIVGDVILGLVCFRMVASEEMNQALLTKLNSSGRIHMVPA
      W. bancrofti  (388)  EQLIRADNIFEIVGDVILGLVCFRMVASEEMNQALLTKLNSSGRIHMVPA
        B. timori   (385)  EQLIRADNIFEIVGDVILGLVCFRMVASEEMNQALLTKLNSSGRIHMVPA
           L. loa   (401)  EQLIRADNIFEIVGDVILGLVCFRMVASEEMNQALLTKLNSSGRIHMVPA
   L. sigmodontis   (401)  EQLIRADNIFEIIGEVILGLVCFRMVASEEMNQELLTKLNSSGRIHMVPA
       O. volvulus  (388)  EQLIRADDIFEIVGDVILGLVCFRMVASEEMNQALLTKLNSSGRIHMVPA
        Consensus   (401)  EQLIRADNIFEIVGDVILGLVCFRMVASEEMNQALLTKLNSSGRIHMVPA
                           451                                              500
        B. malayi   (451)  SLNGRFVIRFCVCAEHATEKDIQIAYDFISQTARHIYQD-SVSCPLAEDE
      W. bancrofti  (438)  SLNGRFVIRFCVCAEHATEKDIQIAYDFISQTARHIYQD-SVSCPLAEDD
        B. timori   (435)  SLNGRFVIQD-----------------SVS-------------CPLAEDE
           L. loa   (451)  SLNGRFVIRFCVCAEHATEKDIQVAYDFISQTARHIYQD-SISCPLAEDD
   L. sigmodontis   (451)  SLNGRFVIRFCVCAEHATERDIQVAYDLISQTARHIYQN-SVSCPLVEDD
       O. volvulus  (438)  SLNGRFVIRFCVCAEHATEKDIRIAYELISQTARHIHQNSLVSYPLVEED
```

```
     Consensus  (451) SLNGRFVIRFCVCAEHATEKDIQIAYDFISQTARHIYQD SVSCPLAEDD
                      501                                            550
      B. malayi (500) ELETAEEKEECIEQISLLAFFFKNKKKEKAFALFRKEKATLSQKRSFLVR
   W. bancrofti (487) ELETAEEK--------------------E-ECIGQIKKATLSQKRSFLVR
      B. timori (455) ELETAEEKE---------ECIEQISLLAFFFKNKKKEKAFALFKRSFLVR
         L. loa (500) ELETAEEKE---------ECIEQITGLNTTEILKRKEQATLSQKRSFLVR
 L. sigmodontis (500) ELETTEGK------------KKDTEQISDTSVCKTQK-------------
    O. volvulus (488) ELGTAEEKG--------CIGQINEEDLNKNEILKRKNQATLSQKRSFLVR
      Consensus (501) ELETAEEKE                Q    L    EILKRKEKATLSQKRSFLVR
                      551                                            600
      B. malayi (550) MVSDPKCYNPKIVRHLTMGGFRKMNEDVVRDLSLREAIGRTKIKVTN---
   W. bancrofti (516) MVSDPKCYNPKIVRHLTMGGFRKMNEDVVRDLSLREAIGRTKIKVTNTNN
      B. timori (496) MVSDPKCYNPKIVRHLTMGGFRKMNEDVVRDLSLREAIGRTKIKVTN---
         L. loa (541) MVSDPKCYNPKIVRHLTMSGFRKMNEDVVRDVSLRETIGRSKIGVT---G
 L. sigmodontis (525) --------------------------------------------------
    O. volvulus (530) MVSDPKCYNPKIVRHLTKSGFRKMNEDVIRDLSLRQTISKTTQNQ-----
      Consensus (551) MVSDPKCYNPKIVRHLTMGGFRKMNEDVVRDLSLREAIGRTKIKVTN
                      601                                            650
      B. malayi (597) LKYFKHDNVNTASSSSGPSVDEEEVIASSNTIRNY---------------
   W. bancrofti (566) MKYFKHDNVNTASSSSGPSVDEEEVIASSNTIRNY---------------
      B. timori (543) LKYFKHDNVNTASSSSGPSVDEEEEQRKNQRPGSLGPVATKWQESSDSDV
         L. loa (588) LKYFKCDNVNTASSSSAPSVEEEVITFPNTTRN-----------------
 L. sigmodontis (525) --------------------------------------------------
    O. volvulus (575) --------------------------------------------------
      Consensus (601) LKYFKHDNVNTASSSSGPSVDEEEV    NT
                      651
      B. malayi (632) ----------
   W. bancrofti (601) ----------
      B. timori (593) QKETQKSNPN
         L. loa (621) ----------
 L. sigmodontis (525) ----------
    O. volvulus (575) ----------
      Consensus (651)
```

Figure S4. Amino acid sequence alignments of potential tyrosine decarboxylase enzymes for *O. volvulus* (Ovo-tdc-1; Bioproject PRJEB513) *L. loa* (LOAG_07708; PRJNA60051), *W. bancrofti* (WBA_0000427401; PRJEB536), *B. malayi* (Bma-tdc-1; PRJNA10729-FR3), *B. timori* (BTMF_0001232601; PRJEB4663), and *L. sigmodontis* (nLs.2.1.2.t05618; PRJEB3075) involved in the biosynthesis of the biomarker *N*-acetyltyramine-*O*-glucuronide (NATOG) from L-tyrosine.

```
                          1                                                 50
      B. malayi     (1) MRCFFHLEGREIIGLLLYIGFQVLSGGDLLWFRIDERMNLRVAKADDLLN
   W. bancrofti     (1) -------------------------------------MNLRVAKADDLLN
         L. loa     (1) -------------------------------------MNLRVAKADDLLN
 L. sigmodontis     (1) --------------MYKTSKIFHLDFDDMQTIQIDERMNLRVAKADDLLN
     O. volvulus    (1) -------------------------------------MNLRVAKADDLLN
      Consensus     (1)                                      MNLRVAKADDLLN
                          51                                               100
      B. malayi    (51) TQHCNLLCLPENYQMKYYFYHALSWPQLSYVAEDDKGNIVGYVLAKMEEE
   W. bancrofti    (14) TQHCNLLCLPENYQMKYYFYHALSWPQLSYVAEDDKGNIVGYVLAKMEEE
         L. loa    (14) TQHCNLLCLPENYQMKYYFYHALSWPQLSYVAEDDKGNIVGYVLAKMEEE
 L. sigmodontis    (37) TQHCNLLCLPENYQMKYYFYHALSWPQLSYVAEDDKGNIVGYVLAKMEEE
     O. volvulus   (14) TQHCNLLCLPENYQMKYYFYHALSWPQLSYVAEDDKGNIVGYVLAKMEEE
      Consensus    (51) TQHCNLLCLPENYQMKYYFYHALSWPQLSYVAEDDKGNIVGYVLAKMEEE
                          101                                              150
      B. malayi   (101) ADDEPHGHITSLAVKRSYRRLGLAQKLMDQTARAMIETFNARYVSLHVRV
   W. bancrofti    (64) ADDEPHGHITSLAVKRSYRRLGLAQKLMDQTARAMIETFNARYVSLHVRV
         L. loa    (64) ADDEPHGHITSLAVKRSYRRLGLAQKLMDQTARAMIETFNARYVSLHVRV
 L. sigmodontis    (87) ADDEPHGHITSLAVKRSYRRLGLAQKLMDQTARAMIETFNARYVSLHVRV
     O. volvulus   (64) ADDEPHGHITSLAVKRSYRRLGLAQKLMDQTARAMVETFNARYVSLHVRV
      Consensus   (101) ADDEPHGHITSLAVKRSYRRLGLAQKLMDQTARAMIETFNARYVSLHVRV
                          151                                              200
      B. malayi   (151) SNRAALNLYQNTLKFEISDVEPKYYADGEDAYAMKRPLVQFAMENNIEPA
   W. bancrofti   (114) SNRAALNLYQNTLKFEISDVEPKYYADGEDAYAMKRPLVQFAMENNIEPA
         L. loa   (114) SNRAALNLYQNTLKFEISDVEPKYYADGEDAYAMKRPLVQFAIENNIEPA
 L. sigmodontis   (137) SNRAALNLYQNTLKFEISDVEPKYYADGEDAYAMKRPLVQFAMENNIEPA
     O. volvulus  (114) SNRAALNLYQNTLKFEISDVEPKYYADGEDAYAMKRPLVQFAMENNIEPA
      Consensus   (151) SNRAALNLYQNTLKFEISDVEPKYYADGEDAYAMKRPLVQFAMENNIEPA
                          201                221
      B. malayi   (201) DRKAFFSTKEEREHARKLKNN
   W. bancrofti   (164) DRKAFFSTKEEREHARKLKNN
         L. loa   (164) DRKAFFSTKEEREHARKQKNN
 L. sigmodontis   (187) DRKAFFSTKEEREHARKQKNN
     O. volvulus  (164) DRKAFFSTKEEREHARKQKNN
      Consensus   (201) DRKAFFSTKEEREHARKQKNN
```

Figure S5. Amino acid sequence alignments of potential *N*-acetyltransferase enzymes for *O. volvulus* (OVOC6155; Bioproject PRJEB513) *L. loa* (LOAG_10487; PRJNA60051), *W. bancrofti* (WBA_0000820201; PRJEB536), *B. malayi* (Bm4892; PRJNA10729-FR3) and *L. sigmodontis* (nLs.2.1.2.t02879; PRJEB3075) involved in the biosynthesis of the biomarker *N*-acetyltyramine-*O*-glucuronide (NATOG) from L-tyrosine.

```
                            1                                                50
        B. malayi     (1)   MAPSENAENSTGDNSNDQYGTHQVNGQSFVINDEPRRVRCVGRCDMELGD
        B. timori     (1)   MAPSENAENSTGDNSNDQYGTHQVNGQSFVINDEPRRVRCVGRCDMELGD
           L. loa     (1)   MAPSENAENAN-DNSKNQRGACEVNGLSFVMKDEPRRVRCVGRCDMELGD
  L. sigmodontis      (1)   MAPSENTENAANDSSKSQCTTCEVNGEPFVIKNEPRQERCVGRCDMELGD
      O. volvulus     (1)   MAPSENAENVANENSKN--Q-CEVNGEPFVMKDEPRQARCVGRCDMELGD
     W. bancrofti     (1)   MAPSENAENSAGDNSNDQCGTHQVNGQSFVINDEPRRVRCVGRCDMELGD
        Consensus     (1)   MAPSENAENSAGDNSNDQ GTHQVNGQSFVINDEPRRVRCVGRCDMELGD
                            51                                              100
        B. malayi    (51)   VTHHNVEQLKRLNQAVFPVAYNDKFYKEIVTAGELAKLAYFNDIVVGGVC
        B. timori    (51)   VTHHNVEQLKRLNQAVFPVAYNDKFYKEIVTAGELAKLAYFNDIVVGGVC
           L. loa    (50)   ITHHNVEQLKRLNQAVFPVAYNDKFYKEIVTAGELAKLAYFNDIVVGGVC
  L. sigmodontis     (51)   ITHHNVEQLRRLNQAVFPVAYNDKFYKEIVTAGELAKLAYFNDIVVGGVC
      O. volvulus    (48)   VTHHNVEQLRRLNQAVFPVAYNDKFYKEIVTAGELAKLAYFNDIVVGGVC
     W. bancrofti    (51)   VTHHNVEQLKRLNQAVFPVAYNDKFYKEIVTAGELAKLAYFNDIVVGGVC
        Consensus    (51)   VTHHNVEQLKRLNQAVFPVAYNDKFYKEIVTAGELAKLAYFNDIVVGGVC
                            101                                             150
        B. malayi   (101)   CRIDTQNGMRRLYIMTLGTLAPYRRHGIGTMLLEHVFTLCDRDPTIENIY
        B. timori   (101)   CRIDTQNGMRRLYIMTLGTLAPYRRHGIGTMLLEHVFTLCDRDPTIENIY
           L. loa   (100)   CRIDTQSGIRRLYIMTLGTLAPYRRLGIGTMLLEHVFTLCDKDPTIENIF
  L. sigmodontis    (101)   CRIDTQNGARRMYIMTLGTLAPYRRLGIGTMLLEHVFTLCNKDPTIEDIY
      O. volvulus    (98)   CRIDTQNGIRRLYIMTLGTLAPYRRLGIGTMLLEHVFVLCDKDPAIDNIY
     W. bancrofti   (101)   CRIDTQNGMRRLYIMTLGTLAPYRRLGIGTLLLEHVFTLCDRDPTIENIY
        Consensus   (101)   CRIDTQNGMRRLYIMTLGTLAPYRRLGIGTMLLEHVFTLCDKDPTIENIY
                            151                                             200
        B. malayi   (151)   LHVQINNESALDFYKRFGFEIVGVAEKYYKRIEPDSAYVLVKKIHREVRE
        B. timori   (151)   LHVQINNESALDFYKRFGFEIVGVAEKYYKRIEPDSAYVLVKKIHREVRE
           L. loa   (150)   LHVQINNESALDFYKRFGFEVVGVAEKYYKRIEPDSAYILVKRIEREVRE
  L. sigmodontis    (151)   LHVQINNESALDFYKRFGFEVAGMAEKYYKRIEPDSAYILVKKIDREVRE
      O. volvulus   (148)   LHVQINNESALDFYKRFGFEVVGIAEKYYKRIEPDSAYILVKKIEREFRD
     W. bancrofti   (151)   LHVQINNESALDFYKRFGFEIVGVAEKYYKRIEPDSAYVLVKKIDREVRE
        Consensus   (151)   LHVQINNESALDFYKRFGFEIVGVAEKYYKRIEPDSAYILVKKIDREVRE
                            201
        B. malayi   (201)   NLP
        B. timori   (201)   NLP
           L. loa   (200)   HLP
  L. sigmodontis    (201)   HLP
      O. volvulus   (198)   HLP
     W. bancrofti   (201)   NLP
        Consensus   (201)   NLP
```

Figure S6. Amino acid sequence alignments of potential *N*-acetyltransferase enzymes for *O. volvulus* (OVOC3302; Bioproject PRJEB513) *L. loa* (LOAG_11415; PRJNA60051), *W. bancrofti* (WBA_0000619201; PRJEB536), *B. malayi* (Bm3964; PRJNA10729-FR3), *B. timori* (BTMF_0001268701; PRJEB4663), and *L. sigmodontis* (nLs.2.1.2.t02879; PRJEB3075) involved in the biosynthesis of the biomarker *N*-acetyltyramine-*O*-glucuronide (NATOG) from L-tyrosine.

```
                                1                                                50
           B. malayi      (1)  MFSEQYILVPLTEKKN----LCSECIELLN-QEWPRSVGARENTLRKSLN
           B. timori      (1)  MFSEQYILVPLTEKKN----LCSECIELLN-QEWPRSVGARENTLRKSLN
        W. bancrofti      (1)  MLNEQYILIPLTKKEN----LCTECIELLN-QEWPRSVGARENTLRKSLN
              L. loa      (1)  -MTAQASMFGWNLKRDDWHFAVNQCYEFYDGQEWPRSVGARENTQRKSLN
      L. sigmodontis      (1)  MFSGQYVLVPLTERKD----LCLECIELLN-QEWPRSIGARENTLRKSLN
         O. volvulus      (1)  MFDEQYIFVPLIERKD----LCSECINLLN-QEWPRSLGARENTLRKSLN
O. volvulus 9342+9343     (1)  MFDEQYIFVPLIERKD----LCSECINLLN-QEWPRSLGARENTLRKSLN
           Consensus      (1)  MFSEQYILVPLTEKKD    LCSECIELLN QEWPRSVGARENTLRKSLN
                                51                                              100
           B. malayi     (46)  SSPPMSFIMVDKKTNELVGHARLCPLLAVSQSCWIESVIIDSNLRGKGLG
           B. timori     (46)  SSPPMSFIMVDKKTNELVGHARLCPLLAVSQSCWIESVIIDSNLRGKGLG
        W. bancrofti     (46)  STPPMSFIMVDKKTDKLVGHARLCPLLAVSQSCWIESVIINRNLRGKGLG
              L. loa     (50)  STPPMSFVMVDKKTDKLVGHGRLCPLLGESQSCWIESVIINNDLRGKGLG
      L. sigmodontis     (46)  SRPPMSFVLVDRNTKKLVGHARLCPIPAISHSCWIESVIINSNLRGKGLG
         O. volvulus     (46)  STPPMSFIVIDKKTDKLIGHARLCPLLTVPQSCWIESVIIDSDLRGKGLG
O. volvulus 9342_9343    (46)  STPPMSFIVIDKKTDKLIGHARLCPLLTVPQSCWIESVIIDSDLRGKGLG
           Consensus     (51)  STPPMSFIMVDKKTDKLVGHARLCPLLAVSQSCWIESVIIDSNLRGKGLG
                                101                                             150
           B. malayi     (96)  CWLMAQLEDEARKFGFKKAYLSSENRQAFYAKCGYATCEPVLNVGANAAL
           B. timori     (96)  CWLMAQLEDEARKFGFKKAYLSSENRQAFYAKCGYATCEPVLNVGANAAL
        W. bancrofti     (96)  YWLMAQLEDEARKFGFKKAYLSSENKQAFYAKCGYTSCEPVLNVGANTAL
              L. loa    (100)  RWLMAQLENEARKFGFRKAYLSSENKQAFYAKCGYSACEPVLNVGVNTAL
      L. sigmodontis     (96)  WWLMAKLEDEARKFGFRKAYLSSKNEQGFYAKCGYSICEPVLNAGANTSL
         O. volvulus     (96)  CWLMNQLENEARKFGFKKIYLSSENKQAFYSKCGYSACEPVLNVGANTVL
O. volvulus 9342_9343    (96)  CWLMNQLENEARKFGFKKIYLSSENKQAFYSKCGYSACEPVLNVGANTVL
           Consensus    (101)  CWLMAQLEDEARKFGFKKAYLSSENKQAFYAKCGYSACEPVLNVGANTAL
                                151                                             200
           B. malayi    (146)  FERFNLGGYFSSAVDGSTSN--------------ERITRINSS-KPSNSC
           B. timori    (146)  FERFNLGGYFSSAVDGSTSN--------------ERITRINSS-KPSNSC
        W. bancrofti    (146)  FERFNLGGYFSSAIDGSTSN--------------ERITRTNSS-KPSDSC
              L. loa    (150)  FERFNLRRCFSSAFDGSTSS--------------ERITGTNSS-KPSSSC
      L. sigmodontis    (146)  LEKFDLGKFFTSAAFGGSIN--------------DRITQTNSSNFSSTSC
         O. volvulus    (146)  FERFDLGRCLLPAFNGSISNRSLSVAMFV---------------------
O. volvulus 9342_9343   (146)  FERFDLGRCLLPAFNGSISNRSLSVAMFVMSGRQERITGINSS-KSNSSG
           Consensus    (151)  FERFNLGRYFSSA DGSTSN              ERIT  NSS KPS SC
                                201                                             250
           B. malayi    (181)  VSALPSEKSLLFPP-----------PAPPPPPPPFGISVLSSGEASKFDG
           B. timori    (181)  VSALPSEKSLLFPP-----------PAPPPPP--FGISVLSSGEASKFDG
        W. bancrofti    (181)  TSALSSEKGLSFSP-----------PTPPPPPPPFRTSVLSSGEASKFDG
```

```
              L. loa        (185) TSALPSEKSLVSPPPAA---------ASPPPPPPPPLASSLSSGEILKFGS
       L. sigmodontis       (182) TSTTSSEKSLPLPP-----------PAPPPPPG-PTSALLPSDEILKSGA
          O. volvulus       (175) --------------------------------------------------
 O. volvulus 9342_9343      (195) MSILSSEKNLAFPSAALSPSLPSLPPPPPPLPPPSLTSKASSAEISKFDS
            Consensus       (201)  SAL SEKSL FPP           PAPPPPPPP   SVLSSGE SKFDG
                                  251
           B. malayi        (220) KKVHYMFKIL
           B. timori        (218) KKVHYMFKIL
         W. bancrofti       (220) KKVHYMFKIL
              L. loa        (227) KKTHYMFKIL
       L. sigmodontis       (220) LQAVFRLY--
          O. volvulus       (175) ----------
 O. volvulus 9342_9343      (245) NKTHYMFKIV
            Consensus       (251) KK HYMFKIL
```

Figure S7. Amino acid sequence alignments of potential *N*-acetyltransferase enzymes for *O. volvulus* (OVOC9342; Bioproject PRJEB513) *L. loa* (LOAG_05435; PRJNA60051), *W. bancrofti* (WBA_0000446201; PRJEB536), *B. malayi* (Bm2986; PRJNA10729-FR3), *B. timori* (BTMF_0000907301; PRJEB4663), and *L. sigmodontis* (nLs.2.1.2.t01383; PRJEB3075) involved in the biosynthesis of the biomarker *N*-acetyltyramine-*O*-glucuronide (NATOG) from L-tyrosine. Entry *O. volvulus* 9342_9343 consist of the gene transcripts OVOC9242 and OVOC9343 combined.

Table S1

Table S1: Detailed NATOG values described in this study (*Figs. 2*, *3, S2, and S3.*).

		c(NATOG) / µM					
Fig. 2 + S2:	N=	Mean value	SEM	Median	Geometric mean	Min value	Max value
***O. volvulus* pos.**	145	42.8	3.7	29.3	23.0	0.9	276.0
**Neg. control**	118	6.4	0.7	3.6	3.8	0.2	39.6
L. loa	100	14.7	2.5	6.8	6.8	0.4	175.6
M. perstans	25	13.6	2.5	11.4	7.9	0.3	46.4
Mp/Ll	3	6.0	2.7	6.5	4.1	1.0	10.4
Ov/Ll	21	16.6	2.8	13.8	10.8	0.8	41.1
Ov/Mp	29	29.2	4.8	17.1	19.0	2.2	92.8
Ov/Ll/Mp	8	100.5	33.5	66.4	52.0	4.7	246.5
Fig. 3 + S3:							
***Ov*-pos. (mono):**	All *Ov*-positive mono-infection samples (This study: N=145 + Ref. 6: *Ov*-pos and Placebo N=95)						
	240	40.3	2.7	26.0	23.2	0.9	276.0
**All *Ov*-pos.:**	All *Ov*-containing samples (This study: N=145 + Ref. 6: N=95 + *Ov/Ll*: N=21 + *Ov/Mp*: N=29 + *Ov/Ll/Mp*: N=8)						
	298	39.2	2.5	24.8	22.0	0.8	276.0
**All *Ov*-neg.:**	All *Ov*-negative samples (This study: N=118 + *L. loa*: N=100 + *M. perstans*: N=25 + *Mp/Ll*: N=3 + Ref 6: Control(Africa+US): N=33 + Ref 6: *Lymph. Fil*: N=23).						
	302	9.29	1.0	4.4	4.4	0.2	175.6

References

1. Wanji, S.; Kengne-Ouafo, J.A.; Esum, M.E.; Chounna, P.W.N.; Tendongfor, N.; Adzemye, B.F.; Eyong, J.E.E.; Jato, I.; Datchoua-Poutcheu, F.R.; Kah, E.; Enyong, P.; Taylor, D.W. *Parasit. Vectors* **2015**, *8*, 202.

2. Arndts, K.; Specht, S.; Debrah, A.Y.; Tamarozzi, F.; Klarmann Schulz, U.; Mand, S.; Batsa, L.; Kwarteng, A.; Taylor, M.; Adjei, O.; Martin, C.; Layland, L.E.; Hoerauf, A. *PLoS Negl. Trop. Dis.* **2014**, *8*, e2679.

3. Wanji, S.; Tayong, D.B.; Layland, L.E.; Datchoua Poutcheu, F.R.; Ndongmo, W.P.C.; Kengne-Ouafo, J.A.; Ritter, M.; Amvongo-Adjia, N.; Fombad, F.F.; Njeshi, C.N.; Nkwescheu, A.S.; Enyong, P.A.; Hoerauf, A. *Parasit. Vectors* **2016**, *9*, 311.

4. Wanji, S.; Amvongo-Adjia, N.; Koudou, B.; Njouendou, A.J.; Chounna Ndongmo, P.W.; Kengne-Ouafo, J.A.; Datchoua-Poutcheu, F.R.; Fovennso, B.A.; Tayong, D.B.; Fombad, F.F.; Fischer, P.U.; Enyong, P.I.; Bockarie, M. *PLoS Negl. Trop. Dis.* **2015**, *9*, e0004184.

5. Debrah, L.B.; Nausch, N.; Opoku, V.S.; Owusu, W.; Mubarik, Y.; Berko, D.A.; Wanji, S.; Layland, L.E.; Hoerauf, A.; Jacobsen, M.; Debrah, A.Y.; Phillips, R.O. *Parasit. Vectors* **2017**, *10*, 15.

6. Globisch, D.; Moreno, A.Y.; Hixon, M.S.; Nunes, A.A.K.; Denery, J.R.; Specht, S.; Hoerauf, A.; Janda, K.D. *Proc. Natl. Acad. Sci. U. S. A.* **2013**, *110*, 4218-4223.
